# A novel cost effective and high-throughput isolation and identification method for marine microalgae

**DOI:** 10.1186/1746-4811-10-26

**Published:** 2014-08-07

**Authors:** Martin T Jahn, Katrin Schmidt, Thomas Mock

**Affiliations:** 1 School of Environmental Sciences, University of East Anglia, Norwich Research Park, Norwich NR4 7TJ, UK; 2 Current address: Department of Botany II, Julius-Maximilians University Würzburg, Julius-von-Sachs-Platz 3, 97082 Würzburg, Germany

**Keywords:** Marine microalgae, Direct PCR, Isolation, Cultivation, Taxonomy

## Abstract

**Background:**

Marine microalgae are of major ecologic and emerging economic importance. Biotechnological screening schemes of microalgae for specific traits and laboratory experiments to advance our knowledge on algal biology and evolution strongly benefit from culture collections reflecting a maximum of the natural inter- and intraspecific diversity. However, standard procedures for strain isolation and identification, namely DNA extraction, purification, amplification, sequencing and taxonomic identification still include considerable constraints increasing the time required to establish new cultures.

**Results:**

In this study, we report a cost effective and high-throughput isolation and identification method for marine microalgae. The throughput was increased by applying strain isolation on plates and taxonomic identification by direct PCR (dPCR) of phylogenetic marker genes in combination with a novel sequencing electropherogram based screening method to assess the taxonomic diversity and identity of the isolated cultures. For validation of the effectiveness of this approach, we isolated and identified a range of unialgal cultures from natural phytoplankton communities sampled in the Arctic Ocean. These cultures include the isolate of a novel marine Chlorophyceae strain among several different diatoms.

**Conclusions:**

We provide an efficient and effective approach leading from natural phytoplankton communities to isolated and taxonomically identified algal strains in only a few weeks. Validated with sensitive Arctic phytoplankton, this approach overcomes the constraints of standard molecular characterisation and establishment of unialgal cultures.

## Background

Marine microalgae are unicellular photosynthetic eukaryotes of major ecological and economic importance worldwide. Ecologically, they are the base of the marine food web and contribute to at least 30% of annual CO_2_ fixation worldwide and therefore massively impact global biogeochemical cycles [1,2]. Economically, diverse marine microalgae are used or have the potential to be used as nutraceuticals, for the production of pharmaceuticals [3,4], cosmetics [5], for bioremediation [6-8], and biofuels [9].

In recent years, the emerging application of culture-independent omics approaches like metagenomics and metatranscriptomics delivered comprehensive insights into the gene repertoire and activity of marine microalgal communities [10-13]. However, results from high-throughput omics approaches ideally need to be scrutinized by experiments with isolated strains from the same communities if the scientific endeavour goes beyond purely describing the diversity and abundance of genes and transcripts in relation to environmental conditions. Similarly, in the field of microalgae biotechnology, novel isolation and identification protocols are essential for identifying specific traits like lipid content [14,15] or any other bioactive compounds [16]. Thus, there is a high demand to develop novel isolation and identification protocols. However, laborious standard procedures such as single-cell isolation of strains, DNA extraction, purification, amplification, sequencing and taxonomic identification include several time, cost and space consuming constraints.

To overcome these constraints, we developed a new cost effective and high-throughput isolation and identification method for marine microalgae. We combined high throughput isolation by streaking cells from enrichment cultures on agar plates with subsequent cultivation in multi-well plates. To assess as to whether a culture was unialgal or not, we applied direct PCR (dPCR) by only using boiling MiliQ water to lyse the cells in combination with a novel sequencing electropherogram based assessment method. While using the V4 as the most variable small subunit (SSU) [17], the underlying idea was that molecular marker sequences of different species possess different bases at the same position. This concept is similar to the detection of intraspecific point mutations exploiting sequencing electropherogram tracefiles [18]. The ambiguous base-calls detected as a biased uncalled/called peak ratio increase the position specific error probability (Pe) [19], which decreases the per-base Quality Values (QV = -10 log_10_(Pe)) as a standard quality metric [20]. The per-base quality values were used in our approach to evaluate the presence or absence of an unialgal culture.

This new approach is relatively cost effective, time saving and high throughput to overcome the constraints of standard molecular characterisation (e.g. by DGGE or RFLP) and establishment of unialgal cultures without the need of DNA extraction and cloning. To validate the efficiency of this approach, we isolated and identified algal strains from natural phytoplankton communities of the Arctic Ocean.

## Results

The objective of this study was to establish a cost and time effective method for microalgal isolation and identification. Using the methods described below, we were able to obtain 24 unialgal cultures consisting of 7 unique ribotypes based on the V4 18S rDNA region.

### Efficiency of growing algae on plates and dPCR

**Figure 1:**
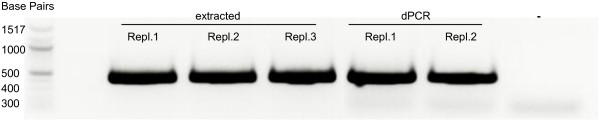
**Comparison of PCR- Products utilizing extracted DNA and direct culture as template.** Kit extracted DNA is amplified in three replicates (Repl.) using the primers TAReuk454FWD1 and TAReukREV3 [36]. Amplification from direct culture (dPCR) in two replicates using same primers as described in the methods section. **–** represents negative control. Whole PCR products (50 μl) are separated and visualised by ethidium bromide staining on a 1% TAE agarose gel.

**Figure 2:**
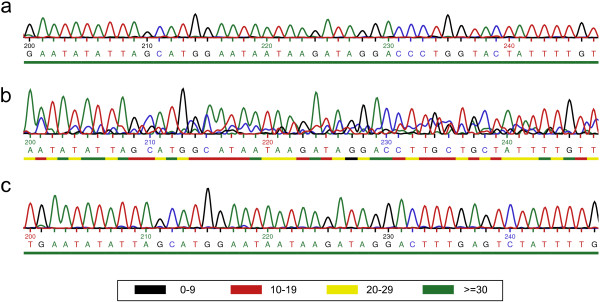
**Representative sequencing electropherogram sections.** Compared are the base calling signal noise of **(a)** unialgal *Thalassiosira pseudonana* laboratory culture **(b)** non-unialgal culture 1-80-15-M with 2 morphospecies **(c)** unialgal classified culture of *Skeletonema marinoi* (2-80-8-M). The color code refers to the per base Quality Values (QV) as the –10 log10(Pe), with Pe as the base call error probability.

Using the high-throughput isolation technique of streaking enriched natural microalgal communities across agar plates, on 59.3% (35 of 59) of the incubated plates algal growth was detected. From about three quarters (77.1%; 27 of 35) of these plates, it was possible to pick single colonies. Moreover, all (158 of 158) of the picked colonies transferred to 12-well plates showed visible growth under the microscope after 1.5 weeks of cultivation. In a preliminary study, primers amplifying the whole 18S rDNA (~1750 bp) region were used for unialgal assessment and taxonomic identification. However, dPCR amplicon sequencing from the 5′ end of the whole 18S rDNA region lacked sufficient variability compared to the V4 sub-region on the 18S rRNA gene. By combining the dPCRs of the whole 18S rDNA and of the V4 region of 18S rDNA, the dPCR approach succeeded in 70.25% (85 of 121) of the reactions. Furthermore, the amplicons obtained by dPCR, as shown in Figure 1, had identical size compared to the control PCR conducted with extracted DNA. Also, no additional bands were visible for dPCRs.About 65% of the screened cultures (24 of 37) were identified as unialgal based on our new electropherogram-based assessment. Figure 2 illustrates the discrimination principle between sequences from unialgal cultures and mixed populations.

### Taxonomy and geographic origin

**Figure 3:**
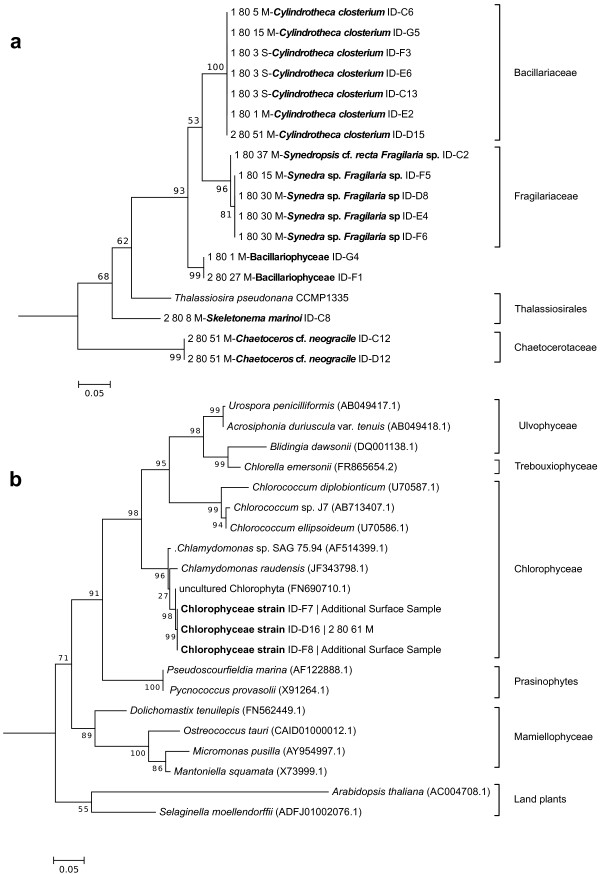
**Maximum-likelihood (ML) trees built from the alignments of V4 18S rDNA sequences.** Molecular phylogeny of **(a)** isolated diatom groups and **(b)** Chlorophyceae with related clades. Nucleotide sequences obtained in the underlying study indicated by species names in bold. Further sequences were obtained from the SILVA database (http://www.arb-silva.de) given with accession numbers. The trees with the highest log likelihood (**(a)** -1355.5135; **(b)** -2321.7603) were inferred using the Maximum Likelihood method based on the Kimura 2-parameter model with MEGA6. The fraction of replicate trees in which the associated taxa clustered together is shown next to the branches (1000 bootstraps). The outgroups were **(a)***Arabidopsis thaliana* and **(b)***Mus musculus*. All positions with less than 80% site coverage were excluded for tree construction. The scale bar represents number of substitutions per site.

**Figure 4:**
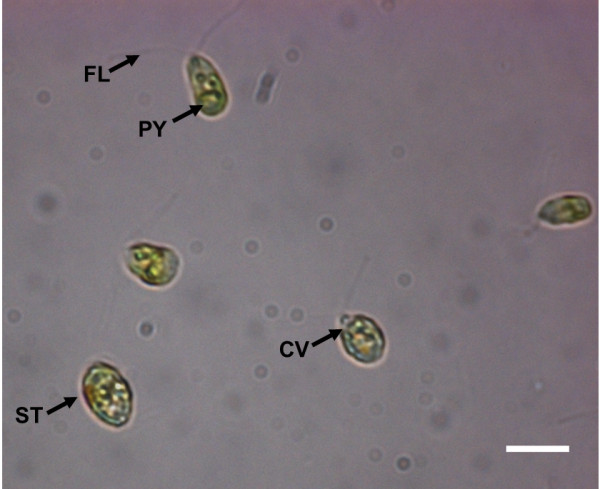
**Phase contrast micrograph of novel Chlorophyceae isolate with isokont flagella (FL), pyrenoid (PY), stigma (ST) and contractile vacuole (CV).** The cells, about 10 μm in size, were grown at 4°C, 24 h day light, 150 μmol photon m^-2^ s^-1^. Magnification 100×, scale bar = 10 μm.

In total, 6 different taxonomic groups were identified based on V4-18S rDNA sequences. NCBI nucleotide BLAST searches (Table 1) revealed that all groups comprised microalgae including an array of 4 different classes with Bacillariophyceae, Fragilariophyceae, Coscinodiscophyceae and Chlorophyceae (Table 1; Figure 3). Noticeable morphological features of the novel Chlorophyceae strain are its contractile vacuoles, two isokont flagella, stigmata, pyrenoids and the size of 10 μm (Figure 4). With the exception of this novel Chlorophyceae strain (Figure 3b), diatoms made up the vast majority of isolated species (Table 1). Amongst diatoms pennate species were twice as often isolated and identified by BLAST searches as centric species, which is in agreement with our microscopic observations (10 of 16 isolates). However, it was found that the V4 region failed to resolve differences within the family Fragilariaceae between the genera *Syndrea*, *Fragilaria* and *Synedropsis* (Table 1, Figure 3a), despite equal sequence quality and length. A similar situation was found in two cultures between the best hits *Nitzschia thermalis* and *Amphora* sp. (1-80-1-M and 2-80-27-M). However, taxonomic groups clustered with high bootstrap support (Figure 3a).

**Table 1:** Closest BLAST matches against NCBI- database of Sequences recovered from isolated Arctic Ocean samples

**SampleID**	**% < QV20**	**Closest species BLAST search hits**	**Last certain common taxonomic assignment**	**Times isolated**	**NCBI Sequence ID**	**Score**	**Expect**	**Identities**	**Gaps**
1-80-1-M	0	Uncultured marine eukaryote	Class: Bacillariophyceae		GU385607.1	599 (324)	3.00E-167	324/324(100%)	0/324(0%)
		Bacillariophyta sp.			KF177731.1	593 (321)	2.00E-165	323/324(99%)	0/324(0%)
		*Nitzschia thermalis*			AY485458.1	588 (318)	7.00E-164	322/324(99%)	0/324(0%)
		*Amphora* sp.			AY485451.1	588 (318)	7.00E-164	322/324(99%)	0/324(0%)
	0	*Cylindrotheca closterium*	Species: *closterium*		HM070405.1	595 (322)	4.00E-166	322/322(100%)	0/322(0%)
1-80-3-S	0- 0.30	*Cylindrotheca closterium*	Species: *closterium*	3	HM070405.1	608 (329)	6.00E-170	329/329(100%)	0/329(0%)
1-80-5-M	0.93	*Cylindrotheca closterium*	Species: *closterium*		HM070405.1	597 (323)	1.00E-166	323/323(100%)	0/323(0%)
2-80-8-M	0	*Skeletonema marinoi* (5)	Species: *marinoi*		HM805045.1	665 (360)	0	360/360(100%)	0/360(0%)
1-80-15-M	0.61	Fragilariaceae sp.	Family: Fragilariaceae		JF794051.1	608 (329)	6.00E-170	329/329(100%)	0/329(0%)
		*Synedra hyperborea* Grunow			HQ912621.1	608 (329)	6.00E-170	329/329(100%)	0/329(0%)
		*Synedra minuscula*			EF423415.1	608 (329)	6.00E-170	329/329(100%)	0/329(0%)
		*Fragilaria* sp.			EU090021.1	608 (329)	6.00E-170	329/329(100%)	0/329(0%)
		*Fragilaria* cf. *striatula*			AJ971377.1	608 (329)	6.00E-170	329/329(100%)	0/329(0%)
	0	*Cylindrotheca closterium*	Species: *closterium*		HM070405.1	595 (322)	4.00E-166	322/322(100%)	0/322(0%)
2-80-27-M	0.30	Uncultured marine eukaryote	Class: Bacillariophyceae		GU385607.1	610 (330)	2.00E-170	330/330(100%)	0/330(0%)
		Bacillariophyta sp.			KF177731.1	604 (327)	7.00E-169	329/330(99%)	0/330(0%)
		*Nitzschia thermalis*			AY485458.1	599 (324)	3.00E-167	328/330(99%)	0/330(0%)
		*Amphora* sp.			AY485451.1	599 (324)	3.00E-167	328/330(99%)	0/330(0%)
1-80-30-M	0- 0.31	Fragilariaceae sp.	Family: Fragilariaceae	3	JF794051.1	595 (322)	4.00E-166	322/322(100%)	0/322(0%)
		*Synedra hyperborea*			HQ912621.1	595 (322)	4.00E-166	322/322(100%)	0/322(0%)
		*Synedra minuscula*			EF423415.1	595(322)	4.00E-166	322/322(100%)	0/322(0%)
		*Fragilaria* sp.			EU090021.1	595 (322)	4.00E-166	322/322(100%)	0/322(0%)
		*Fragilaria* cf. *striatula*			AJ971377.1	595 (322)	4.00E-166	322/322(100%)	0/322(0%)
1-80-37-M	0.31	*Synedropsis* cf. *recta*	Family: Fragilariaceae		HQ912616.1	584 (316)	1.00E-162	318/319(99%)	0/319(0%)
		*Fragilaria striatula*			EU090018.1	584 (316)	1.00E-162	318/319(99%)	0/319(0%)
		*Fragilaria barbararum*			AJ971376.1	584 (316)	1.00E-162	318/319(99%)	0/319(0%)
2-80-51-M	0- 0.62	*Chaetoceros* cf. *neogracile*	cf. species: *neogracile*	2	JN934684.1	595 (322)	4.00E-166	322/322(100%)	0/322(0%)
	0.55	*Cylindrotheca closterium*	Species: *closterium*		HM070405.1	667 (361)	0	361/361(100%	0/361(0%)
2-80-61-M	0.94	Uncultured Chlorophyta	Class: Chlorophyceae		FN690710.1	582 (315)	3.00E-162	317/318(99%)	0/318(0%)
		*Chlamydomonas raudensis*			AJ781313.1	555 (300)	7.00E-154	312/318(98%)	0/318(0%)
NA	0	*Cylindrotheca closterium*	Species: *closterium*		HM070405.1	606 (328)	2.00E-169	328/328(100%)	0/328(0%)
	0	*Synedropsis* cf. *recta*	Family: Fragilariaceae		HQ912616.1	636 (344)	3.00E-178	346/347(99%)	0/347(0%)
		*Fragilaria striatula*			EU090018.1	636 (344)	3.00E-178	346/347(99%)	0/347(0%)
		*Fragilaria barbararum*			AJ971376.1	636 (344)	3.00E-178	346/347(99%)	0/347(0%)
	0	*Cylindrotheca closterium*	Species: *closterium*		HM070405.1	665 (360)	0	360/360(100%)	0/360(0%)
	0.62	*Skeletonema marinoi*	Species: *marinoi*		HM805045.1	597 (323)	1.00E-166	323/323(100%)	0/323(0%)
	0	Uncultured Chlorophyta	Class: Chlorophyceae	2	FN690710.1	588 (318)	7.00E-164	320/321(99%)	0/321(0%)
		*Chlamydomonas raudensis*			AJ781313.1	560 (303)	2.00E-155	315/321(98%)	0/321(0%)

**Figure 5:**
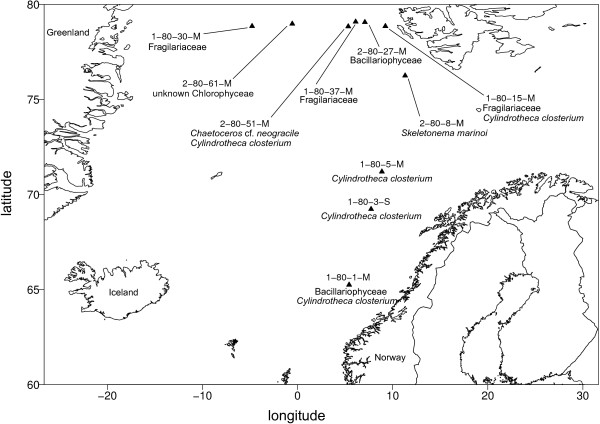
**Arctic Ocean Map representing distribution of isolated algae.** Each ▲ denotes a sampling point with hyphen separated unique identifier (trial-Polarstern cruise number- Cruise stop number-depth [S = Surface/M = Chlorophyll max]) and closest certain taxonomy according to a BLAST search against GeneBank.

Using our approach, we were most successful in isolating the pennate diatom *Cylindrotheca closterium* 9 times from a variety of 5 different sampling locations along most of the latitudinal transect (latitude: 65.246- 78.839) of this study (Figure 5). The Fragilariaceae-cluster (Figure 3a) in contrast was only recovered as an isolate from samples originating from the northernmost sites (Figure 5). On the west side of the transect, a novel Chlorophyceae was isolated from the chlorophyll maximum in a depth of 10 m. *Chaetoceros* cf. *neogracile* and *Skeletonema marinoi* were collected from location 2-80-51-M and 2-80-8-M, respectively (Figure 5).

## Discussion

In recent years, huge efforts were made to establish culture collections holding thousands of marine algae strains like in the National Center for Marine Algae and Microbiota (NCMA). Novel approaches of cryopreservation [21,22] reduced culture maintenance efforts considerably [23]. This study reports an approach that enabled us to establish a range of unialgal cultures from Arctic Ocean samples (1) under cost effective conditions due to the omission of DNA-extraction and cloning (2) with low space requirements due to the use of 12-well format (3) within processing times of three weeks.

In accordance with previous studies, the isolated species *Cylindrotheca closterium*[24], *Skeletonama marinoi*[25] and *Chaetoceros* cf. *neogracile*[26] were already identified in the Arctic Ocean and are available in culture collections. The given morphological features of the novel Chlorophyceae strain together with the clustering of its V4 18S rDNA ribotype into *Chlamydomonas* may indicate closer affiliation towards this genus. Even though several different *Chlamydomonas* species were identified in Antarctic saline lakes [27,28], on Arctic glaciers [29], or in sea ice of the brackish Baltic Sea [30], this would be, to our knowledge, the first record of a marine *Chlamydomonas* strain from the deep Chlorophyll maximum layer in the open ocean. However, further characterisation of this strain is needed what is beyond the scope of this methodical paper. It remains to be seen how significant marine Chlorophyceae species are in terms of diversity, abundance and activity in relation to members of the class Prasinophyta.

Every isolation method has biases towards specific groups to be successfully isolated. Plating, as our method of choice, was reported to exclude some flagellates and coccoids and most dinoflagellates [23]. Alternatively, combining dPCR with other isolation techniques like single-cell sorting [31,32] may increase the spectrum of isolated strains and especially those that won’t grow well on agar plates. However, the costs of single-cell isolation and its biases (e.g. selection against filamentous and larger algae) seem to object to our approach.

The success rates of our dPCR approach clearly emphasise the advantages of using microalgae cultures as they grow without the need of DNA extraction as described previously [33-35]. However, a limitation of dPCR might be the use of the V4 region. Nevertheless, the V4 region used as a molecular marker in this study represents the most variable SSU region [17]. However, dinoflagellates possess less variability in this region [36] making it more difficult to taxonomically characterise isolates without ambiguity. Despite the fact that we had longer reads (average 361 bp) available for BLAST searches against NCBI compared with Stoeck et al. [36] (average 270 bp), it was still not sufficient to resolve taxonomies within Fragilariaceae and between *Nitzschia* sp. and *Amphora* sp.. In fact, the V4- region as a molecular marker was found to be too conserved to allow taxonomic resolution in these cases.

The use of sequencing electropherograms for analytical purposes like the detection of point mutations [18] or multiple clone sequences [37] is frequently reported. In our case, using the novel electropherogram based analysis allowed distinction between sequences from a single strain/species and sequences from multiple strains/species. A crucial step is the formulation of a well-defined algorithm for an objective trimming of the sequences. The requirements in this context are twofold. On one hand, sequences from unialgal cultures have to be trimmed at regular drops of quality at the end and the beginning of the sequence reads. On the other hand, sequences from mixed communities containing low quality reads should only be trimmed to a distinct lower length limit for a reliable assessment as described in the methods section. We expect that interspecific length polymorphisms of the V4 region increase the sensitivity of our culture assessment due to the fact that only one base shift would lead to a screwed sequence.

## Conclusions

Our method is suitable for establishing unialgal cultures from mixed natural communities within a few weeks on a cost effective and high-throughput basis. Further improvements could include isolation on low-meting agar for sensitive species such as flagellates, picking of algal colonies from plates with robots and cultivation in 96-well plates under various conditions (e.g. different media, light and temperature) to increase the likelihood of isolating rare species or strains.

## Materials and methods

### Study sites and sample collection

For the low cost and high throughput isolation and identification of marine arctic microalgae a total of 27 water samples was taken along a latitudinal gradient (65.25°N to 79.37°N) from the Arctic Ocean during June and July 2012. Briefly, 12 L seawater was sampled either at the chlorophyll maximum (23 samples; depths 7-110 m) or at the surface (4 samples; depth 5 m) using a Niskin bottle rosette sampler. Additionally, at each sampling depth, temperature, salinity, surface irradiance as well as chlorophyll and nutrient concentration (NO_3_, NH, PO_4_, Silicate) were measured (see Additional file 1). Sea water was pre-filtered through a 100 μm mesh and the flow-through fraction (<100 μm) was transferred into f/2-medium [38] for enrichment of natural microalgal communities. Whilst transferred regularly into fresh medium, the samples were enriched cultured 425 days at 4°C and about 150 μmol photon m^-2^ s^-1^ for ca. 50 generations before unialgal cultures were isolated. However, the time for enrichment is variable depending on the temperature-dependent growth rates of the algal communities.

### High throughput microalgae isolation

Isolation of microalgae into unialgal cultures was done by streaking the enriched microalgal communities across agar plates as described previously [23]. In short, environmental sample cultures were plated on chilled petri dishes containing f/2-medium solidified with 1% (w/v) agar. Subsequently, the agar plates were incubated at 4°C, 24 h day cycle, 150 μmol photon m^-2^ s^-1^ in a light thermostat (Rumed, Rubarth Apparate GmbH, Laatzen, Germany) for 1-2 weeks. Clearly separated colonies were picked from the plates at the end of the striping and transferred each to 3 ml fresh f/2-medium provided in space efficient 12- well plates. Plates without clearly separated colonies where discarded. Inoculated 12-well plates were incubated for 1.5 weeks at 4°C, 24 h day light, 150 μmol photon m^-2^ s^-1^ to increase cell density. These cultures were screened for a) the presence of algae cells (fluorescence emission from chlorophyll a) and b) for visual inspection of having unialgal cultures based on uniform morphology of at least 200 individual algal cells using a phase contrast microscope at 400× maginfication (Olympus BX40, Olympus Optical Co., Ltd., Japan) equipped with Olympus Camedia C-7070 wide-zoom digital camera. Cultures that met both criteria were kept for further molecular analysis.

### Direct polymerase chain reaction

For the direct PCR (dPCR)- amplification of ribosomal DNA, a volume of 500 μl suspended culture from each of the positive wells according to the visual inspection criteria (see above) was transferred to 1.5 ml centrifuge tubes and incubated for 5 min at 100°C (Dry Bath Heating System, Starlab, Milton Keynes, United Kingdom) to inactivate protease activity. Then algal cells were harvested by centrifugation at 16,000 rpm for 10 min at room temperature (Eppendorf centrifuge 5418 R, Germany) and the supernatant was discarded. In order to disrupt the algal cell integrity the pellet was re-suspended properly with 100 μl boiling MiliQ-water. The 4°C chilled suspension was either used directly for PCR or stored at -20°C until further use.

Primers TAReuk454FWD1 (5′-CCAGCA(G⁄C)C(C⁄T)GCGGTAATTCC-3′) and TAReukREV3 (5′-ACTTTCGTTCTTGAT(C⁄T)(A⁄G)A-3′) [36] were used to amplify the V4- region of the 18S rDNA using TC-512 PCR System (Techne Co. Staffs, UK). The dPCR was carried out in 50 μL reaction tubes with 10 μl prepared suspension as template, 2.5 U/μl Taq DNA polymerase (GoTaq® Flexi DNA polymerase, Promega, Madison, WI, USA), 1× Taq reaction Buffer, 2 mM MgCl2, 0.2 mM each dNTP, and 0.4 μM of each primer. The parameters of thermal cycling of Stoeck et al. (2010) [36] were slightly modified to 30 s initial denaturation at 98°C, 10 × (98°C, 10 s; 53°C, 30 s; 72°C, 30 s), 20 × (98°C, 10 s; 48°C, 30 s; 72°C, 30 s) and 10 min final extension at 72°C.

### Gel purification and sequencing

The dPCR-products were visualised on 1% (w/v) TAE-agarose gels stained with ethidium bromide. Amplicon bands of the expected size of 421 bp (*Fragilariopsis cylindrus*) were cut and gel purified using the NucleoSpin® Gel and PCR Clean-up kit (Macherey-Nagel GmbH & Co. KG, Düren, Germany) according to the manufacturer’s instructions. The DNA yield and purity of the purified dPCR-products were evaluated using the NanoDrop ND-1000 spectrophotometer (NanoDrop Technologies, Wilmington, USA). Finally, utilising the TA-Reuk454FWD1 forward primer, the amplicons were Sanger-sequenced on a ABI 3730XL sequencer by Eurofins MWG Operon (Ebersberg, Germany).

### Nucleotide sequence analysis

The sequencing chromatogram trace (.ab1- format) was analysed and trimmed using the ABI Sequence Scanner v1.0 (Applied Biosystems™). Sequence trimming as well as evaluation of the unialgal status was based on implemented per-base Quality Values (QV) as –10 log_10_(Pe), with Pe as the base call error probability [19]. These QV consider chromatogram features like peak spacing, uncalled/called peak ratio and peak resolution. The sequences were trimmed: a) at the 5′end after the first 25-35 bp when the QV consecutive was >20 in a 20 bp window and b) at the 3′end starting after 350 bases, before the first 20 consecutive basecalls contained more than 1 bases with < QV20. Whilst taking the sequencing machine basecalling accuracy of 98.5% [39] into account, the trimmed sequences were classified as unialgal, when the fraction of <20QV basecalls was smaller than one percent. For taxonomic identification BLAST sequence similarity searches [40] of as unialgal classified cultures against the NCBI database (http://www.ncbi.nlm.nih.gov; release 199) were performed using the megablast algorithm. Multiple sequence alignments of the obtained V4 18S rDNA-sequences were done using ClustalX v1.6 [41] and curated using Gblocks v0.91b [42]. A rooted phylogenetic tree was produced by MEGA v6.0 [43] using the maximum likelihood method based on the Kimura 2-parameter model [44] excluding positions with less than 80% site coverage. The robustness of the inferred tree was estimated using a bootstrap analysis consisting of 1000 resampling’s of the data.

The nucleotide sequences have been deposited in GenBank and a representative set of cultures was deposited in the Culture Collection of Algae and Protozoa (CCAP) under accession numbers given in Additional file 2.

## Competing interests

The authors declare that they have no competing interests.

## Authors’ contributions

The experiments were conceived by MTJ, KS and TM and performed by MTJ. The data was analysed by MTJ. KS performed microscopy of the Chlorophyceae strain and collected the samples. MTJ and TM co-wrote the paper. All authors read and approved the final manuscript.

## Supplementary Material

Additional file 1 Metadata of study sites. Click here for file

Additional file 2 Culture accession numbers of this study. Click here for file
