# Supplementary material for: A novel cost effective and high-throughput isolation and identification method for marine microalgae
Source: Plant Methods. 2014 Aug 7;10:26. doi: 10.1186/1746-4811-10-26 (PMC4128616; doi:10.1186/1746-4811-10-26)
Supplement: Additional file 1 — Metadata of study sites. [file 1746-4811-10-26-S1.pdf]

Additional files

A1: Metadata of study sites

Station	Date	lat	long	Depth	T at depth	Salinity at depth	Chlorophyll at depth	W/m2	NO3 [µM]	NH [µM]	PO4 [µM]	Si [µM]
PS80/1-S	17.06.12	65.24611	5.41917	5	9.75	34.94	1.4	87	1.58	1.18	0.59	2.35
PS80/1-M	17.06.12	65.24611	5.41917	20	9.24	34.93	1	87				
PS80/2-S	17.06.12	67.23028	6.53028	20	8.6781	35.1	0.55	548	2.38	1.54	0.66	2.12
PS80/2-M	17.06.12	67.23028	6.53028	15	8.9384	35.091	0.58	548				
PS80/3-S	18.06.12	69.23028	7.73028	5	8.7545	34.8661	0.9	51	0.86	1.11	0.49	1.68
PS80/3-M	18.06.12	69.23028	7.73028	10	9.0976	34.7321	0.9	51				
PS80/5-M	18.06.12	71.20083	8.86667	10	7.1834	35.1344	0.9	64	4.83	0.79	0.77	3.62
PS80/7-M	19.06.12	73.01889	9.85667	20	6.0186	35.1528	0.8	58	6.55	0.78	0.88	3.90
PS80/8-M	19.06.12	76.25389	11.30917	15	5.5282	35.1514	1.6	172	6.26	0.89	0.83	4.08
PS80/15-M	20.06.12	78.85139	9.23306	25	2.3374	34.7579	5.5	277	2.93	2.75	0.94	2.16
PS80/20-M	20.06.12	78.86972	8.11222	10	5.269	35.0693	0.9	56	0.51	1.47	0.49	2.75
PS80/27-M	21.06.12	79.06667	7.07639	15-20	4.5614	35.0886	1.8	43	9.69	0.78	1.07	4.31
PS80/37-M	22.06.12	79.08944	6.10556	7	4.8199	35.1105	3	38	3.90	0.89	0.76	3.88
PS80/51-M	24.06.12	78.83889	5.32861	15	4.4939	35.0994	3.5	251	3.32	0.62	0.73	3.95
PS80/55-S	25.06.12	79.07278	3.73583	5	-0.3027	33.1465	2.5	111	2.21	0.36	0.51	3.12
PS80/55-M	25.06.12	79.07278	3.73583	10	0.0522	33.586	3	111				
PS80/61-M	26.06.12	78.98167	-0.55917	10	1.3665	33.5306	1	346	0.69	0.51	0.50	3.64
PS80/63-M	27.06.12	78.93667	-1.84	16	4.3973	35.062	7	63	4.77	0.79	0.84	3.26
PS80/87-M	01.07.12	78.89667	-2.83722	25	-1.3686	33.3389	3.5	68	0.70	0.15	0.46	3.03
PS80/87-M	01.07.12	78.89667	-2.83722	110	3.0802	34.9887	0.5	68				
PS80/118-M	06.07.12	79.0225	-9.52472	17	-1.0337	31.0274	0.9	79	0	0.25	0.47	2.48
PS80/120-M	07.07.12	79.07611	-8.52472	26	-1.5122	31.708	0.6	38	0.14	0.26	0.95	2.86
PS80/122-M	07.07.12	79.04278	-7.67278	20	-1.4645	31.3282	1.8	111	0	0.20	0.62	7.17
PS80/130-M	08.07.12	78.85611	-4.78556	20	-1.6191	32.2039	1.9	105	0.60	0.36	0.74	8.97
PS80/132-M	09.07.12	79.00056	-4.08556	10	-1.5083	32.9517	2	115	0.81	0.55	0.58	5.49
PS80/132-M	09.07.12	79.00056	-4.08556	35	-1.7398	33.9123	1.2	115				
PS80/135-M	09.07.12	78.86694	-3.22861	15	-0.8805	32.3454	1.6	117	3.30	0.49	0.79	5.47
