# Supplementary material for: A novel cost effective and high-throughput isolation and identification method for marine microalgae
Source: Plant Methods. 2014 Aug 7;10:26. doi: 10.1186/1746-4811-10-26 (PMC4128616; doi:10.1186/1746-4811-10-26)
Supplement: Additional file 2 — Culture accession numbers of this study. [file 1746-4811-10-26-S2.pdf]

A2: Culture accession numbers of this study

Sample ID	Name	GenBank accession number	Culture Collection of Algae and Protozoa (CCAP) deposition number
ID-C1	NA- Fragilariaceae	KM197158	
ID-C12	2 80 51 M-*Chaetoceros* cf. *neogracile*	KM197152	CCAP 1010/40
ID-C13	1 80 3 S-*Cylindrotheca closterium*	KM197141	
ID-C2	1 80 37 M-*Synedropsis* cf. *recta Fragilaria* sp.	KM197144	CCAP 1029/28
ID-C4	NA-*Cylindrotheca closterium*	KM197159	
ID-C6	1 80 5 M-*Cylindrotheca closterium*	KM197137	
ID-C8	2 80 8 M-*Skeletonema marinoi*	KM197151	CCAP 1077/12
ID-D12	2 80 51 M-*Chaetoceros* cf. *neogracile*	KM197153	CCAP 1010/39
ID-D15	2 80 51 M-*Cylindrotheca closterium*	KM197143	CCAP 1017/15
ID-D16	NA-Chlorophyceae strain	KM197154	
ID-D8	1 80 30 M-*Synedra* sp. *Fragilaria* sp	KM197146	
ID-E2	1 80 1 M-*Cylindrotheca closterium*	KM197142	
ID-E4	1 80 30 M-*Synedra* sp. *Fragilaria* sp	KM197147	
ID-E6	1 80 3 S-*Cylindrotheca closterium*	KM197140	
ID-F1	2 80 27 M-Bacillariophyceae	KM197150	
ID-F3	1 80 3 S-*Cylindrotheca closterium*	KM197139	
ID-F5	1 80 15 M-*Synedra* sp. *Fragilaria* sp.	KM197145	CCAP 1029/29
ID-F6	1 80 30 M-*Synedra* sp. *Fragilaria* sp.	KM197148	CCAP 1029/30
ID-F7	NA-Chlorophyceae strain	KM197156	
ID-F8	NA-Chlorophyceae strain	KM197155	
ID-G2	NA-*Skeletonema marinoi*	KM197160	
ID-G4	1 80 1 M-Bacillariophyceae	KM197149	
ID-G5	1 80 15 M-*Cylindrotheca closterium*	KM197138	
ID-G6	NA-*Cylindrotheca closterium*	KM197157	
